# Supplementary material for: International gestational age-specific centiles for blood pressure in pregnancy from the INTERGROWTH-21st Project in 8 countries: A longitudinal cohort study
Source: PLoS Med. 2021 Apr 27;18(4):e1003611. doi: 10.1371/journal.pmed.1003611 (PMC8112691; doi:10.1371/journal.pmed.1003611)
Supplement: S1 Text — (DOCX) [file pmed.1003611.s002.docx]

**S1 Text. Sample Size Calculation**

The sample size for this study was determined by the requirements for the Fetal Growth Longitudinal Study (FGLS), part of the INTERGROWTH‐21^st^ Project. These have been described previously. The following text and table are taken from: <https://obgyn.onlinelibrary.wiley.com/doi/full/10.1111/1471-0528.12031>

Due to the complexity of determining the required sample size for growth reference studies a WHO Expert committee, as early as 1995, recommended, as a rule of thumb, a sample of at least 200 individuals in each age group and sex. However, the concept of age groups does not apply to a fetal growth study like FGLS as all the data will be considered in a single analysis, and fetal sex differences will not be explored. In clinical practice fetal sex is not always determined during pregnancy and therefore growth charts have not been gender specific, especially as. this information is never divulged in some cultures. Also, it has been shown that the differences in birth weight between males and females are very small and we expect the differences in fetal growth during pregnancy to be negligible anyway. Therefore, the minimal sample size per site was calculated without taking fetal sex or age group into account.

We considered the sample size for FGLS in relation to the precision and accuracy of a single centile and regression based reference limits, as first proposed by Royston and extended by Bellera and Hanley. Fetal size measurements tend to be close to a normal distribution at each specific gestational age. Data that are normally distributed can be summarised using the mean and standard deviation from which each required centile can be estimated.

The standard error of the P^th^ centile is given by the standard formula for sampling variance of a centile of normal distribution:


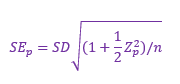


where SE is the standard error, SD is the standard deviation of the measurement (which will increase with gestational age), *Z_p_* is the value of the standard normal distribution corresponding to the P^th^ centile, and n is the sample size. So, for example, for the 2.5th or 97.5th centile *Z_p_* = 1.96, giving SE = 0.08SD for a sample size of 500 and 0.03SD for 4000 fetuses (Table S1). Our sample size calculation was based on a cross‐sectional design and, bearing in mind that the distance between the 2.5th and 97.5th centiles is about 4SD, it is clear that even at these extreme centiles, fetal size will be estimated with great precision with 500 fetuses per site. These standard errors are, however, overestimated as they ignore the fact that there will be a series of measurements from each fetus. Sample size calculations for growth standards based on longitudinal data are complicated and supported by a rather limited literature. Royston defined a design factor, D, as the number of fetuses in a cross‐sectional study that would give the same precision as a longitudinal study. Using ultrasound based biparietal diameter the design factor was suggested to be approximately 2.3. Based on that value, the longitudinal component of FGLS with 4000 fetuses would have equivalent precision to a cross‐sectional study of over 9000 fetuses.

**Table A.** A summary table relating sample size to precision expressed in SD at selected centiles

| **Sample size** | **Precision achieved at 2.5th or 97.5th centile in SD** | **Precision achieved at 5th or 95th centile in SD** | **Precision achieved at 10th or 90th centile in SD** |
| --- | --- | --- | --- |
| 500 | 0.08 | 0.07 | 0.06 |
| 1000 | 0.05 | 0.05 | 0.04 |
| 1500 | 0.04 | 0.04 | 0.03 |
| 2000 | 0.04 | 0.03 | 0.03 |
| 2500 | 0.03 | 0.03 | 0.03 |
| 3000 | 0.03 | 0.03 | 0.02 |
| 3500 | 0.03 | 0.03 | 0.02 |
| 4000 | 0.03 | 0.02 | 0.02 |
| 4500 | 0.03 | 0.02 | 0.02 |
| 5000 | 0.02 | 0.02 | 0.02 |
| 5500 | 0.02 | 0.02 | 0.02 |
| 6000 | 0.02 | 0.02 | 0.02 |
