# Supplementary material for: International gestational age-specific centiles for blood pressure in pregnancy from the INTERGROWTH-21st Project in 8 countries: A longitudinal cohort study
Source: PLoS Med. 2021 Apr 27;18(4):e1003611. doi: 10.1371/journal.pmed.1003611 (PMC8112691; doi:10.1371/journal.pmed.1003611)

**S3 Text. Supporting information**

**Baseline characteristics by site**

**Table A in S3 Text.** Baseline characteristics for women enrolled in the Fetal Growth Longitudinal Study.

|  | Brazil  (n=411) | China  (n=609) | India  (n=625) | Italy  (n=509) | Kenya  (n=617) | Oman  (n=599) | UK  (n=640) | USA  (n=311) | FGLS Cohort (n=4321) |
| --- | --- | --- | --- | --- | --- | --- | --- | --- | --- |
| Age (years) (SD) | 28·3 (4·1) | 26·8 (2·9) | 27·3 (3·5) | 29·0 (4·3) | 28·9 (3·5) | 27·2 (3·8) | 30·2 (3·7) | 29·8 (3·6) | 28·4 (3·9) |
| Body mass index (kg/m^2^) (SD) | 24·2 (2·8) | 22·2 (2·6) | 22·4 (3·0) | 23·0 (2·8) | 24·0 (3·0) | 23·7 (3·2) | 23·3 (2·8) | 23·8 (3·1) | 23·3 (3·0) |
| Gestational age at first visit (weeks) (SD) | 11·5 (1·4) | 12·8 (0·9) | 11·6 (1·5) | 11·9 (1·0) | 11·5 (1·5) | 10·9 (1·4) | 12·3 (1·1) | 11·6 (1·8) | 11·8 (1·4) |
| Years of formal education (years) (SD) | 13·6 (3·3) | 14·2 (2·2) | 16·1 (1·3) | 13·5 (4·0) | 15·5 (1·4) | 14·3 (2·5) | 16·0 (3·0) | 17·1 (2·8) | 15·0 (2·8) |
| Haemoglobin concentration <15 weeks (g/dL) (SD) | 12·4 (0·9) | 13·5 (0·9) | 11·6 (0·6) | 12·9 (0·9) | 12·9 (1·3) | 11·7 (1·1) | 12·7 (0·9) | 12·7 (0·9) | 12·5 (1·1) |
| Married or cohabiting (%) | 393 (95·6) | 607 (99·7) | 619 (99·0) | 493 (96·9) | 558 (90·4) | 599 (100·0) | 632 (98·8) | 303 (97·4) | 4204 (97·3) |
| Nulliparous (%) | 301 (73·2) | 588 (96·6) | 443 (70·9) | 328 (64·4) | 385 (62·4) | 328 (54·8) | 378 (59·1) | 204 (65·6) | 2955 (68·4) |

|  | N | Mean DBP (SD) | SSD | Mean SBP (SD) | SSD |
| --- | --- | --- | --- | --- | --- |
| 9-13^+6^ weeks | | | | | |
| Brazil | 408 | 71·90 (7·874) | 0·334 | 114·8 (10·323) | 0·339 |
| China | 607 | 69·28 (6·709) | -0·049 | 108·9 (8·593) | -0·307 |
| India | 645 | 69·34 (6·862) | -0·041 | 110·5 (8·737) | -0·124 |
| Italy | 508 | 66·78 (7·891) | -0·419 | 111·7 (10·255) | 0·009 |
| Kenya | 606 | 71·03 (7·672) | 0·218 | 112·8 (10·951) | 0·138 |
| Oman | 599 | 69·61 (7·473) | 0·001 | 109·3 (9·742) | -0·262 |
| UK | 629 | 70·00 (7·973) | 0·062 | 114·5 (11·507) | 0·327 |
| USA | 250 | 68·54 (8·151) | -0·148 | 111·1 (10·531) | -0·054 |
| All | 4252 | 69·60 (7·635) | 0 | 111·6 (10·281) | 0 |
| 14-19^+6^ weeks | | | | | |
| Brazil | 401 | 70·52 (8·097) | 0·174 | 116·0 (11·472) | 0·292 |
| China | 566 | 70·62 (7·189) | 0·196 | 112·9 (10·484) | -0·011 |
| India | 554 | 66·91 (7·431) | -0·332 | 107·6 (10·992) | -0·545 |
| Italy | 495 | 64·87 (7·744) | -0·613 | 109·8 (10·194) | -0·317 |
| Kenya | 562 | 69·75 (8·037) | 0·073 | 112·2 (10·618) | -0·078 |
| Oman | 574 | 69·98 (7·987) | 0·106 | 114·7 (11·446) | 0·171 |
| UK | 582 | 71·74 (7·472) | 0·359 | 118·2 (11·059) | 0·526 |
| USA | 339 | 69·26 (9·439) | 0·002 | 112·6 (12·223) | -0·034 |
| All | 4073 | 69·24 (8·131) | 0 | 113·0 (11·464) | 0 |
| 19-23^+6^ weeks | | | | | |
| Brazil | 404 | 69·19 (7·497) | 0·024 | 115·1 (11·064) | 0·162 |
| China | 574 | 71·76 (7·621) | 0·399 | 115·4 (9·939) | 0·193 |
| India | 566 | 66·48 (7·535) | -0·37 | 107·8 (10·686) | -0·596 |
| Italy | 488 | 64·94 (7·601) | -0·581 | 111·1 (9·431) | -0·245 |
| Kenya | 568 | 70·06 (8·250) | 0·151 | 112·4 (10·807) | -0·118 |
| Oman | 592 | 69·05 (6·904) | 0·005 | 115·0 (10·662) | 0·154 |
| UK | 579 | 71·63 (7·364) | 0·38 | 118·9 (11·108) | 0·56 |
| USA | 303 | 67·94 (8·934) | -0·146 | 111·4 (11·474) | -0·204 |
| All | 4074 | 69·02 (7·980) | 0 | 113·5 (11·105) | 0 |
| 24-28^+6^ weeks | | | | | |
| Brazil | 395 | 69·47 (7·277) | -0·055 | 115·3 (10·299) | 0·147 |
| China | 539 | 73·18 (7·393) | 0·484 | 116·4 (10·013) | 0·262 |
| India | 581 | 67·83 (7·396) | -0·299 | 109·0 (10·607) | -0·512 |
| Italy | 472 | 65·58 (7·192) | -0·611 | 110·9 (9·939) | -0·295 |
| Kenya | 572 | 70·48 (7·946) | 0·091 | 112·5 (9·990) | -0·141 |
| Oman | 593 | 69·54 (7·273) | -0·048 | 114·7 (11·111) | 0·089 |
| UK | 580 | 72·23 (7·210) | 0·349 | 118·5 (11·176) | 0·487 |
| USA | 287 | 69·95 (9·936) | 0·013 | 113·3 (13·679) | -0·054 |
| All | 4019 | 69·86 (7·929) | 0 | 113·8 (11·152) | 0 |
| 29-33^+6^ weeks | | | | | |
| Brazil | 407 | 71·23 (7·380) | 0·02 | 116·7 (10·726) | 0·246 |
| China | 659 | 73·95 (7·434) | 0·43 | 116·7 (10·104) | 0·266 |
| India | 555 | 68·94 (7·458) | -0·312 | 108·9 (10·447) | -0·556 |
| Italy | 472 | 67·04 (7·532) | -0·574 | 111·4 (9·914) | -0·291 |
| Kenya | 568 | 71·68 (7·818) | 0·088 | 112·2 (10·344) | -0·216 |
| Oman | 580 | 70·00 (7·336) | -0·158 | 114·7 (10·600) | 0·05 |
| UK | 584 | 74·09 (7·273) | 0·442 | 119·2 (11·493) | 0·515 |
| USA | 304 | 69·99 (9·160) | -0·149 | 113·3 (11·965) | -0·094 |
| All | 4129 | 71·08 (7·941) | 0 | 114·2 (11·115) | 0 |
| 34-40^+6^ weeks | | | | | |
| Brazil | 436 | 75·32 (8·391) | 0·159 | 119·7 (11·866) | 0·267 |
| China | 583 | 76·80 (8·200) | 0·365 | 118·5 (10·512) | 0·151 |
| India | 521 | 71·07 (8·158) | -0·402 | 111·8 (11·097) | -0·507 |
| Italy | 468 | 69·89 (8·142) | -0·551 | 113·1 (10·836) | -0·375 |
| Kenya | 627 | 73·70 (8·118) | -0·053 | 114·6 (10·735) | -0·235 |
| Oman | 675 | 73·21 (7·741) | -0·122 | 117·2 (10·493) | 0·022 |
| UK | 770 | 77·74 (7·697) | 0·518 | 122·2 (11·463) | 0·549 |
| USA | 317 | 72·40 (9·443) | -0·214 | 116·0 (12·261) | -0·089 |
| All | 4397 | 74·09 (8·537) | 0 | 117·0 (11·586) | 0 |

**All site SSD and sensitivity analysis excluding potential outliers**

**Table B in S3 Text.** All sites and individual site means (SD) for diastolic blood pressure (DBP) and systolic blood pressure (SBP) of all women.**Fig A in S3 Text.** Plot to illustrate sensitivity analysis for excluding potential site outliers; systolic blood pressure (SBP) and diastolic blood pressure (DBP) according to gestational age for 3^rd^, 50^th^ and 97^th^ centiles.


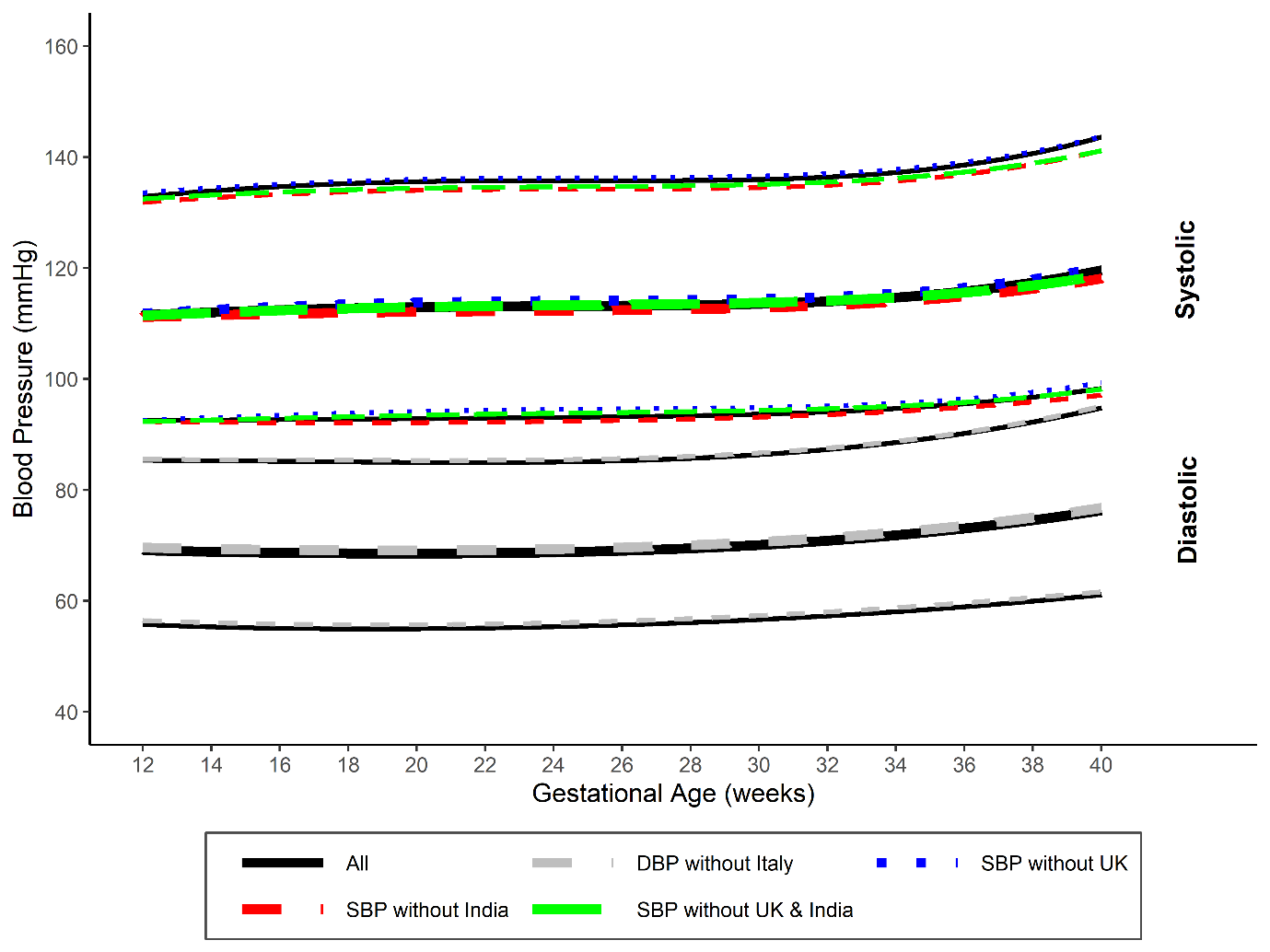


**Values for smoothed centiles for systolic and diastolic blood pressure**

**Table C in S3 Text.** Smoothed centiles for systolic blood pressure (SBP) and diastolic blood pressure (DBP) according to gestational age for 3^rd^, 10^th^, 50^th^, 90^th^ and 97^th^ centiles (95% CI).

|  | Centiles for Systolic Blood Pressure (95% CI) | |  |  |  |
| --- | --- | --- | --- | --- | --- |
| GA | **3rd** | **10th** | **50th** | **90th** | **97th** |
| 12 | 92·56 (92·07, 93·04) | 98·80 (98·42, 99·18) | 111·5 (111·3, 111·8) | 125·6 (125·2, 126·0) | 132·9 (132·4, 133·4) |
| 14 | 92·60 (92·16, 93·04) | 98·99 (98·67, 99·30) | 112·0 (111·8, 112·2) | 126·4 (126·1, 126·7) | 133·9 (133·4, 134·4) |
| 16 | 92·67 (92·26, 93·08) | 99·17 (98·88, 99·45) | 112·4 (112·2, 112·6) | 127·1 (126·7, 127·4) | 134·7 (134·2, 135·1) |
| 18 | 92·75 (92·36, 93·14) | 99·32 (99·05, 99·60) | 112·7 (112·5, 113·0) | 127·6 (127·2, 127·9) | 135·2 (134·7, 135·7) |
| 20 | 92·85 (92·46, 93·23) | 99·46 (99·17, 99·74) | 112·9 (112·7, 113·2) | 127·8 (127·5, 128·2) | 135·6 (135·1, 136·0) |
| 22 | 92·94 (92·56, 93·33) | 99·56 (99·27, 99·86) | 113·1 (112·8, 113·3) | 128·0 (127·7, 128·3) | 135·7 (135·3, 136·2) |
| 24 | 93·05 (92·67, 93·44) | 99·66 (99·36, 99·97) | 113·1 (112·9, 113·4) | 128·1 (127·7, 128·4) | 135·8 (135·3, 136·3) |
| 26 | 93·19 (92·81, 93·58) | 99·78 (99·48, 100·08) | 113·2 (113·0, 113·5) | 128·1 (127·7, 128·4) | 135·8 (135·2, 136·3) |
| 28 | 93·38 (93·00, 93·76) | 99·94 (99·65, 100·24) | 113·3 (113·1, 113·6) | 128·1 (127·7, 128·5) | 135·8 (135·2, 136·4) |
| 30 | 93·66 (93·29, 94·04) | 100·21 (99·92, 100·50) | 113·6 (113·3, 113·8) | 128·3 (127·8, 128·8) | 136·0 (135·3, 136·7) |
| 32 | 94·08 (93·72, 94·44) | 100·63 (100·34, 100·91) | 114·0 (113·7, 114·3) | 128·7 (128·2, 129·3) | 136·4 (135·6, 137·1) |
| 34 | 94·68 (94·32, 95·05) | 101·26 (100·97, 101·55) | 114·7 (114·4, 115·0) | 129·5 (129·0, 130·0) | 137·2 (136·5, 138·0) |
| 36 | 95·54 (95·09, 95·99) | 102·20 (101·85, 102·55) | 115·8 (115·5, 116·1) | 130·8 (130·3, 131·3) | 138·6 (137·9, 139·3) |
| 38 | 96·72 (96·01, 97·43) | 103·52 (102·99, 104·04) | 117·4 (117·0, 117·8) | 132·7 (132·1, 133·4) | 140·7 (139·7, 141·6) |
| 40 | 98·29 (97·07, 99·52) | 105·30 (104·42, 106·19) | 119·6 (118·9, 120·3) | 135·4 (134·2, 136·6) | 143·6 (142·0, 145·2) |

|  | Centiles for Diastolic Blood Pressure (95% CI) | | |  |  |
| --- | --- | --- | --- | --- | --- |
| GA | **3rd** | **10th** | **50th** | **90th** | **97th** |
| 12 | 55·76 (55·45, 56·08) | 59·94 (59·71, 60·18) | 69·14 (68·94, 69·33) | 79·92 (79·59, 80·25) | 85·35 (84·90, 85·81) |
| 14 | 55·32 (55·00, 55·63) | 59·55 (59·33, 59·77) | 68·86 (68·69, 69·02) | 79·77 (79·44, 80·11) | 85·28 (84·82, 85·74) |
| 16 | 55·07 (54·72, 55·41) | 59·32 (59·07, 59·57) | 68·67 (68·49, 68·85) | 79·64 (79·30, 79·98) | 85·17 (84·71, 85·63) |
| 18 | 54·96 (54·60, 55·32) | 59·22 (58·96, 59·47) | 68·56 (68·38, 68·74) | 79·53 (79·21, 79·85) | 85·06 (84·63, 85·49) |
| 20 | 54·98 (54·63, 55·34) | 59·22 (58·98, 59·47) | 68·54 (68·37, 68·70) | 79·46 (79·18, 79·75) | 84·97 (84·60, 85·35) |
| 22 | 55·11 (54·78, 55·45) | 59·33 (59·11, 59·55) | 68·60 (68·45, 68·74) | 79·47 (79·22, 79·71) | 84·95 (84·63, 85·27) |
| 24 | 55·35 (55·03, 55·66) | 59·54 (59·34, 59·74) | 68·76 (68·63, 68·89) | 79·58 (79·37, 79·79) | 85·03 (84·76, 85·31) |
| 26 | 55·68 (55·37, 55·98) | 59·86 (59·66, 60·05) | 69·05 (68·92, 69·18) | 79·83 (79·63, 80·04) | 85·27 (85·00, 85·54) |
| 28 | 56·11 (55·80, 56·42) | 60·29 (60·09, 60·48) | 69·48 (69·34, 69·61) | 80·26 (80·04, 80·48) | 85·70 (85·40, 86·00) |
| 30 | 56·64 (56·32, 56·97) | 60·84 (60·63, 61·05) | 70·07 (69·92, 70·22) | 80·90 (80·64, 81·15) | 86·36 (86·01, 86·70) |
| 32 | 57·29 (56·95, 57·62) | 61·53 (61·30, 61·75) | 70·85 (70·68, 71·01) | 81·78 (81·51, 82·06) | 87·29 (86·92, 87·67) |
| 34 | 58·05 (57·71, 58·39) | 62·36 (62·13, 62·59) | 71·84 (71·66, 72·01) | 82·95 (82·68, 83·22) | 88·55 (88·18, 88·93) |
| 36 | 58·94 (58·58, 59·30) | 63·35 (63·10, 63·61) | 73·06 (72·85, 73·26) | 84·44 (84·16, 84·73) | 90·18 (89·80, 90·57) |
| 38 | 59·95 (59·47, 60·44) | 64·51 (64·15, 64·88) | 74·54 (74·26, 74·83) | 86·31 (85·90, 86·71) | 92·24 (91·70, 92·78) |
| 40 | 61·10 (60·30, 61·90) | 65·85 (65·25, 66·46) | 76·32 (75·88, 76·75) | 88·59 (87·86, 89·32) | 94·77 (93·79, 95·76) |

**Fig B in S3 Text.** Smoothed centiles for systolic blood pressure (SBP) and diastolic blood pressure (DBP) in mmHg; 3^rd^, 10^th^, 50^th^, 90^th^ and 97^th^ centiles with corresponding 95% confidence intervals.


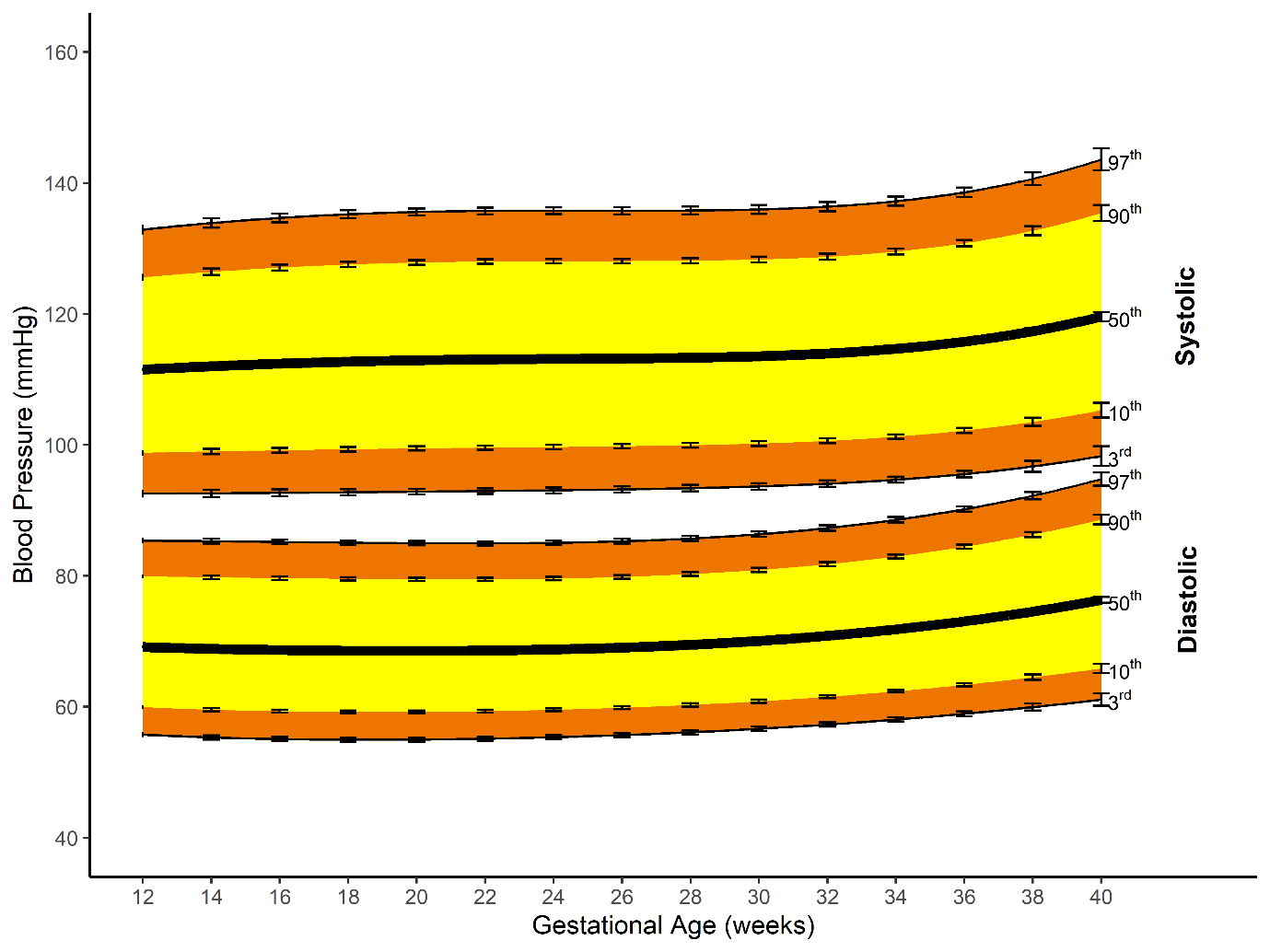


**Smoothed centiles excluding women who developed hypertension**

**Fig C in S3 Text.** Smoothed centiles for systolic blood pressure (SBP) and diastolic blood pressure (DBP) in mmHg excluding women (n=132) who developed hypertension (systolic BP ≥140 or diastolic BP ≥90); 3^rd^, 10^th^, 50^th^, 90^th^ and 97^th^ centiles.


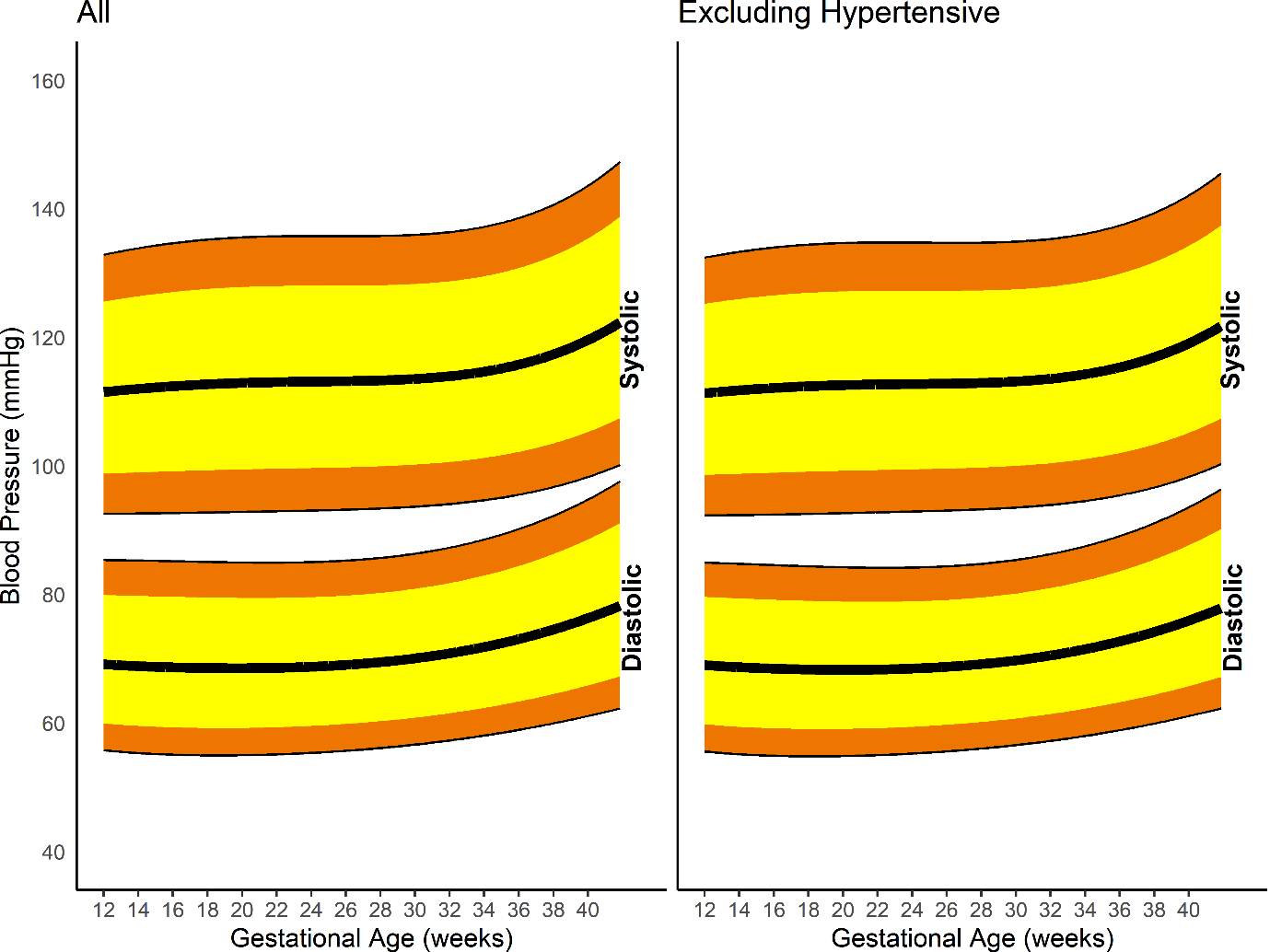


**Blood pressure by quartile of baseline blood pressure and change in systolic and diastolic blood pressure from baseline blood pressure at study enrolment**

**Fig D in S3 Text**. Systolic blood pressure (SBP) and diastolic blood pressure (DBP) from 16 weeks’ gestation onwards for quartiles according to baseline blood pressure (3^rd^, 10^th^, 50^th^, 90^th^ and 97^th^ centiles). First quartile 76-105/40-64 mmHg; second quartile 106-111/65-70 mmHg; third quartile 112-119/71-75 mmHg; fourth quartile ≥120/76 mmHg).


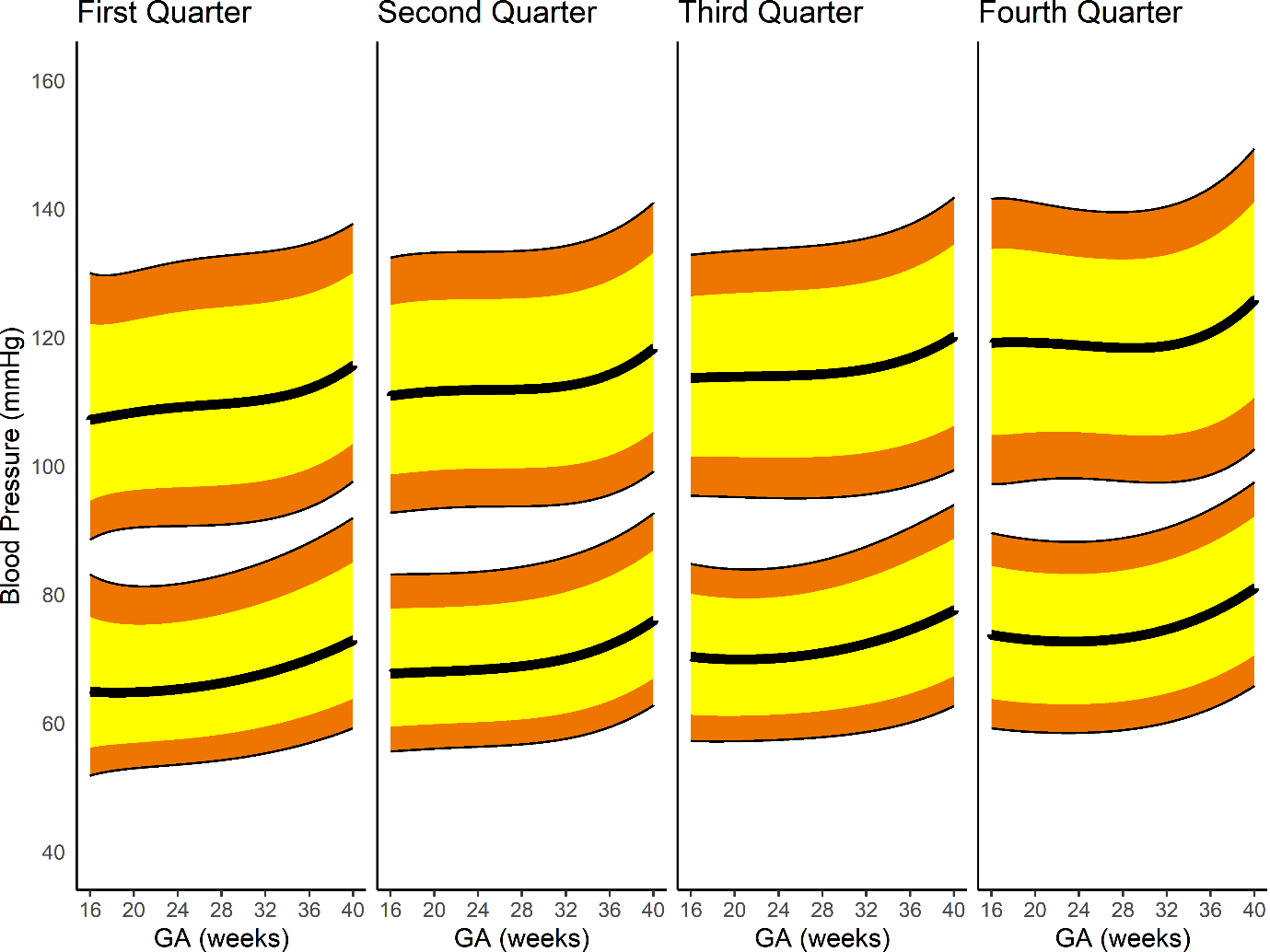


**Fig E in S3 Text.** Change in systolic and diastolic blood pressure during pregnancy by quartiles of baseline blood pressure at study entry at 9-13^+6^ weeks’ gestation.


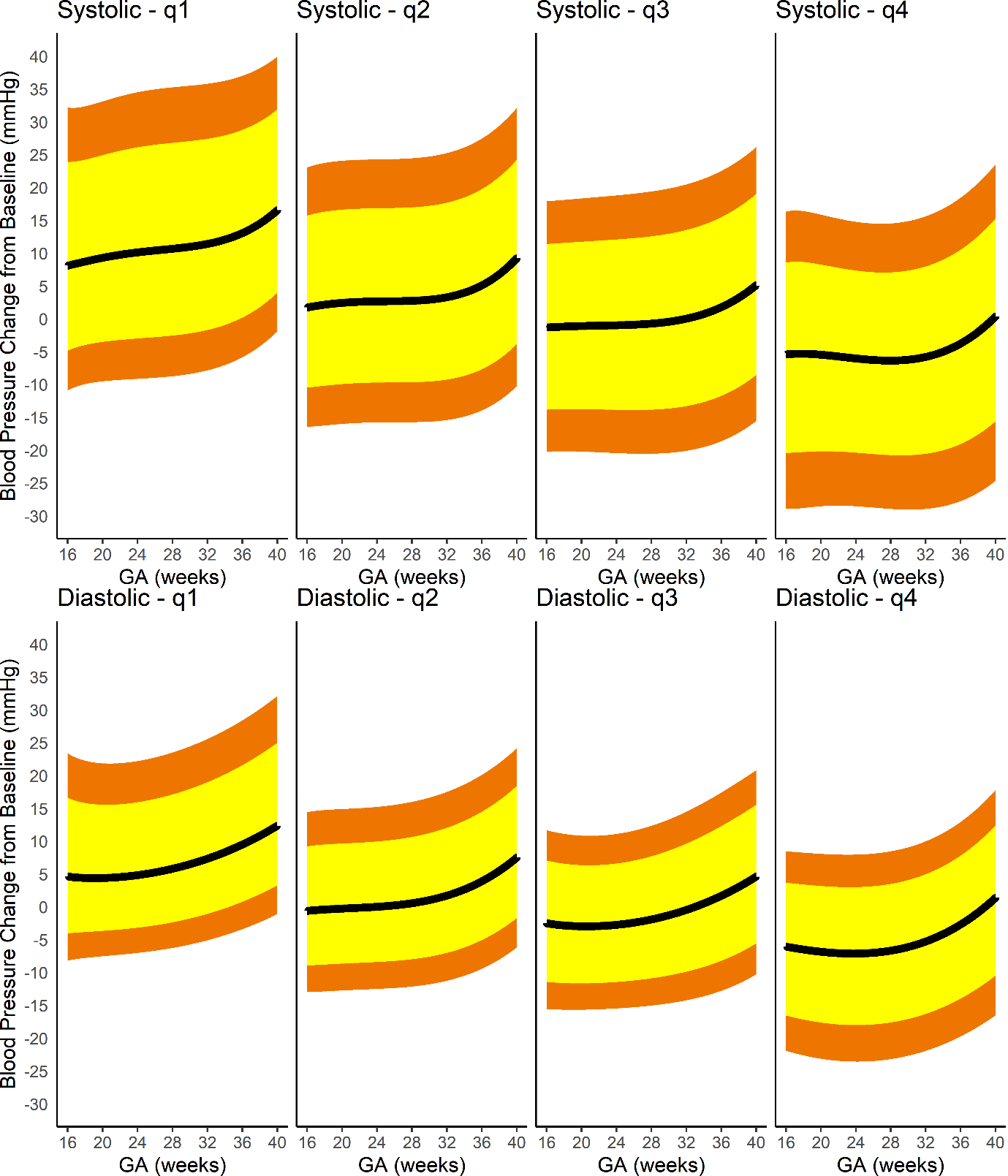

Supplement: S3 Text — Table A in S3 Text: Baseline characteristics for women enrolled in the FGLS. Table B in S3 Text: All sites and individual site means (SD) for DBP and SBP of all women. Fig A in S3 Text: Plot to illustrate sensitivity analysis for excluding potential site outliers; SBP and DBP according to gestational age for third, 50th, and 97th centiles. Table C in S3 Text: Smoothed centiles for SBP and DBP according to gestational age for third, 10th, 50th, 90th, and 97th centiles (95% CI). Fig B in S3 Text: Smoothed centiles for SBP and DBP in mmHg; third, 10th, 50th, 90th, and 97th centiles with corresponding 95% confidence intervals. Fig C in S3 Text: Smoothed centiles for SBP and DBP in mmHg excluding women (n = 132) who developed hypertension (systolic BP ≥140 or diastolic BP ≥90); third, 10th, 50th, 90th, and 97th centiles. Fig D in S3 Text: SBP and DBP from 16 weeks’ gestation onwards for quartiles according to baseline blood pressure (third, 10th, 50th, 90th, and 97th centiles). First quartile 76–105/40–64 mmHg; second quartile 106–111/65–70 mmHg; third quartile 112–119/71–75 mmHg; fourth quartile ≥120/76 mmHg). Fig E in S3 Text: Change in systolic and DBP during pregnancy by quartiles of baseline blood pressure at study entry at 9–13+6 weeks’ gestation. DBP, diastolic blood pressure; FGLS, Fetal Growth Longitudinal Study; SBP, systolic blood pressure; SD, standard deviation; SSD, standardised site difference. (DOCX) [file pmed.1003611.s004.docx]
