# Supplementary material for: International gestational age-specific centiles for blood pressure in pregnancy from the INTERGROWTH-21st Project in 8 countries: A longitudinal cohort study
Source: PLoS Med. 2021 Apr 27;18(4):e1003611. doi: 10.1371/journal.pmed.1003611 (PMC8112691; doi:10.1371/journal.pmed.1003611)
Supplement: S4 Text — INTERGROWTH-21st, International Fetal and Newborn Growth Consortium for the 21st Century. (DOCX) [file pmed.1003611.s005.docx]

**S4 Text. Contributions to membership of the International Fetal and Newborn Growth Consortium for the 21^st^ Century (INTERGROWTH-21^st^) and its Committees**

**Scientific Advisory Committee**

C Garza

**Steering Committee**

ZA Bhutta (Chair), J Villar (Principal Investigator), S Kennedy (Project Director), DG Altman, FC Barros, E Bertino, M Carvalho, L Cheikh Ismail, MG Gravett, YA Jaffer, A Lambert, JA Noble, RY Pang, AT Papageorghiou, M Purwar, C Victora.

**Executive Committee**

J Villar (Chair), DG Altman, ZA Bhutta, L Cheikh Ismail, S Kennedy, A Lambert, JA Noble, AT Papageorghiou.

**Project Coordinating Unit**

J Villar (Head), S Kennedy, L Cheikh Ismail, A Lambert, AT Papageorghiou, EO Ohuma.

**Data Analysis Group**

DG Altman (Head), EO Ohuma, J Villar.

**Data Management Group**

DG Altman (Head), EO Ohuma (from September 2010).

**Ultrasound Group**

AT Papageorghiou (Head), M Carvalho, JA Noble.

**Anthropometry Group**

L Cheikh Ismail (Head), ZA Bhutta.

**Neonatal Group**

ZA Bhutta (Head), E Bertino, RY Pang.

**Participating countries and local investigators**

**Brazil:** FC Barros (Principal Investigator).

**China:** RY Pang (Principal Investigator).

**India:** M Purwar (Principal Investigator).

**Italy:** E Bertino (Principal Investigator).

**Kenya:** M Carvalho (Co- Principal Investigators).

**Oman:** YA Jaffer (Principal Investigator).

**UK:** S Kennedy (Principal Investigator), L Cheikh Ismail, AT Papageorghiou, A Lambert, EO Ohuma.

**USA:** MG Gravett (Principal Investigator).
