# Supplementary material for: International gestational age-specific centiles for blood pressure in pregnancy from the INTERGROWTH-21st Project in 8 countries: A longitudinal cohort study
Source: PLoS Med. 2021 Apr 27;18(4):e1003611. doi: 10.1371/journal.pmed.1003611 (PMC8112691; doi:10.1371/journal.pmed.1003611)
Supplement: Attachment — Submitted filename: PLOS_Medicine_comments_210215.docx [file pmed.1003611.s006.docx]

**Reviewer comments:**

Abstract:

The authors have performed a unique analysis generating gestational age-specific centiles using blood pressure data from a population-based study including women from across the world. Their results are important as they provide information on international standards. Moreover, these results provide insight into physiological blood pressure changes in pregnancy. Overall, this is a clearly designed and very well-written study and the fact that it is prospective adds value to the data. The study uses strong statistical methods and analyses.

I only do have some minor comments:

Minor:

- Abstract:

Results section: Systolic blood pressure: …. , difference (95% CI) 8.1 (7.4 to 8.8) mmHg. This sentence is unclear. At least add the word “a” as is written in the results section. However, the authors preferably state more clearly only a difference in systolic blood pressure of 8.1 mmHg (95% CI: 7.4-8.8) between 12 weeks’ gestation and 40 weeks’ gestation is found.

- Description results:

I suggest to adapt the description of the results to: Median (then value) and subsequent (95% CI: values) which is easier to read and then understand the results.

- Conclusions:

The conclusion that these data provide a strong basis for constructing… (line) is not supported by these data. Hence, in the abstract the authors should use describe it as accordingly in the main paper-discussion: (Line 480-482): These gestational age-specific centiles should help build an….

Results:

Line 355: minimum to maximum difference. The authors should also refer to and add the gestational age then: is it second to third trimester?

Discussion:

Interpretation:

Line 408-409: These findings should aid clinicians in recognizing when a pregnant woman is becoming unwell.

I disagree with this sentence as these findings as for now only help to recognize deviations from normal values.

The authors should change this interpretation of their data as they have not investigated that their values aid in recognizing when a pregnant woman is becoming unwell.

Furthermore, the phrase becoming unwell is too vague. They should state more specifically what they mean by this word.

Additionally, they should state more clearly that it still needs to be thoroughly investigated these values are of additional value for the detection of clinically ill women. Then only, subsequently, as they mention in their conclusion the results will help build an MOEWS to allow earlier recognition.

Limitations:

With the aim of this study to describe blood pressure patterns in a healthy pregnancy women (with an increased risk of ) developing complications are excluded. The authors succeeded as for example a low incidence of gestational hypertension and preeclampsia is reported. They furthermore demonstrated in a sensitivity analysis that exclusion of these women did not affect their results.

However, they should mention in their limitations that the strict inclusion criteria (e.g age 18-35 years, BMI < 30) as described in their methods may contribute to less generalizability of their gestational age-specific centiles for blood pressure. Specifically, taking their future conclusion into account as their results should help clinicians determine patients with abnormal blood pressure rise and falls.

Limitations:

The authors indeed mention that lack of pre-pregnany data may disguise first-trimester changes. They however then jump to the following conclusion (line 453-454): comparison with blood pressure in early pregnancy has greater clinical applicability.

I think this statement is overrated and this conclusion cannot be drawn as there was no comparison with pre-pregnancy data as these are lacking.

Hence, suggest to change this sentence to: in routine clinical practice, blood pressure in early pregnancy contributes to clinical applicability.
