# Supplementary material for: International gestational age-specific centiles for blood pressure in pregnancy from the INTERGROWTH-21st Project in 8 countries: A longitudinal cohort study
Source: PLoS Med. 2021 Apr 27;18(4):e1003611. doi: 10.1371/journal.pmed.1003611 (PMC8112691; doi:10.1371/journal.pmed.1003611)
Supplement: Attachment — Submitted filename: Response to Editors Letter.docx [file pmed.1003611.s007.docx]

Nuffield Department of Clinical Neurosciences,

University of Oxford

15th March 2021

Dear Editors,

Thank you for the opportunity to revise our manuscript in light of the reviewers’ comments. We believe their suggestions have improved our manuscript and are grateful for the thoroughness of the reviews. We have included a point-by-point response, below, which we are happy for you to publish.

Yours faithfully,

Dr Lauren Green

Nuffield Department of Clinical Neurosciences, University of Oxford

Professor Peter Watkinson

Nuffield Department of Clinical Neurosciences, University of Oxford

Requests from the editors: 
 
*Please adapt the study descriptor in your title to refer to the present analysis rather than the parent study. If this analysis is a retrospective analysis of prospectively-gathered data, please remove the word "prospective" from the title.*

Removed 
 
*Please combine the "Methods" and "Results" subsections of your abstract. The final sentence of the new combined subsection should begin "Study limitations include ..." or similar, and should summarize 2-3 of the study's main limitations.*

Thank you. The “Methods” and “Results” have been merged and a sentence on the study’s main limitations has been added. 
 
*Please quote some additional information on participant demographics in the abstract.*

Done 
 
*Please trim the "Conclusions" subsection of your abstract, which should summarize the study's conclusions (claims about "the first" and "the largest" can be removed).*

Done

*After the abstract, please remove the "Research in context" information and substitute a new and accessible "Author summary" section in non-identical prose. You may find it helpful to consult one or two recent research papers in PLOS Medicine to get a sense of the preferred style.*

Done 
 
*Please indicate in the Methods section whether the present analysis had a protocol or prespecified analysis plan, and if so attach the document as a supplementary file, referred to in the text. Please highlight analyses that were not prespecified.*

We included an analysis plan in our application to use INTERGROWTH-21^st^ Project data. We have included this in the Methods section, highlighted analyses that were not pre-specified in the text and included it as new S2 appendix. 
 
*Please restructure the start of the Discussion section so that the first paragraph provides a summary of the study's main findings.*

Done 
 
*Throughout the text, please adapt reference call-outs as follows: "... mid-trimester [11,23]."*. 

Done

*Please remove information on funding and competing interests from the title page and from the end of the main text. In the event of publication, this information will appear in the article metadata, via entries in the submission form.*  

Done

*Please ensure that all references contain full access information, e.g., reference 3.*

Done  
 
*Please supply a completed STROBE checklist with your revision as a supplementary file, labelled "S1_STROBE_Checklist" or similar and referred to as such in your methods section. In the checklist, please refer to individual items by section (e.g., "Methods") and paragraph number rather than by line or page numbers, as the latter generally change in the event of publication.*

Done 
 
Comments from the reviewers: 
 
*** Reviewer #1:  
 
Comments: 
 
*This study is reported to follow STROBE guidelines.  Can the authors please supply a copy of their STROBE checklist in the supplementary material?*

Done 
 
*Given the extent of the relevant supporting documentation, in terms of protocols, data collection forms, and electronic transfer strategies, it is perhaps appropriate that the authors site where readers can access these (*[*www.intergrowth21.org*](http://www.intergrowth21.org/)*), rather than include copies within the supplementary material.*

Thank you, we have followed this suggestion.
 
*"Statistical considerations focused on the precision and accuracy of a single centile and regression based reference limits. FGLS established an average sample of 500 pregnant women per study site, after exclusion of complicated pregnancies (approximately 3%) and those lost to follow-up (estimated to be 3%). This sample size was adequate to explore site-specific differences (18). "* 
*Can more detail on these sample size calculations please be provided by the authors in brief within the supplementary material?*

Done, new S1 appendix included 
 
*"We constructed International, gestational age-specific, smoothed centiles for blood pressure, i.e. 3rd, 10th, 50th, 90th and 97th centiles with corresponding 95% confidence intervals, following WHO recommendations (19). "* 
*The analytical approach applied is robust and rigorous, with a summary of techniques provided in the Methods section.*  
*The statistical methodology is technically appropriate and recommended practice for the data type, research question and study context in hand, including that applied for the subgroup and post hoc analyses.*

Thank you 
 
*** Reviewer #2:  
 
*I appreciated reviewing this manuscript, which represents an important topic of interest in prenatal/antenatal care. The INTERGROWTH-21st Study has brought very critical evidence to obstetrics, and this manuscript promises to continue this trend. However, I have some concerns about it in its current form.* 
 
*1. Throughout the paper, you've used the term "mid-trimester". It may be a case of minor linguistic differences, but to me this term means "in the middle of any trimester" and not in the middle of a specific trimester. At one point you do use "mid-second trimester" and it would be clearer to an international audience to use this phrasing each time.*

Thank you for this wording suggestion – we have changed all references to “mid-pregnancy” 
 
*2. The Research in Context section is meant for general audiences, but is currently very jargony. I think that the Introduction and Discussion of the paper use more accessible language that could be paraphrased here.*

Changed in line with the editors suggestions above. 
 
*3. In the Introduction, the MOEWS needs to be briefly described where it is introduced.*

We have removed the reference to MOEWS in the abstract in light of other reviewers comments 
 
*4. The Methods section is in need of reorganization. All of the subsections that are about the participants (Participants, Bias, Study size) should be together, and all of the sections that are about the analyses (Variables, Quantitative variables, [neither of which, I would argue, describe variables], Other analyses, and Statistical methods) should be together. After rearranging, the grouped subsections will need to be edited to remove redundancy and to more clearly describe the flow of your analysis plan as it is presented in the Results and Supplements.*

We have reorganised the section and reduced redundancy whilst maintaining the structure recommended by STROBE. We sympathise with the reviewer’s view of “quantitative variables” – but note that we have “explained how quantitative variables were handled in the analyses” as recommended in the STROBE checklist 
 
*5. The figure designs need improvement. The symbols representing the Standardized Site Differences in Figure 2 are difficult to discern and need to be enlarged. Also in Figure 2, the SBP plot should go before the DBP plot to be consistent with the title and the presentation of the findings. In many of the remaining figures, SBP and DBP are not clearly distinguished from each other. The color scheme for most of the figures (3, 4, S3-5.2) is not particularly aesthetic, and it is not clear to me why color blocks were used instead of lines.*

Thank you. We have improved symbol size and put SBP first. We have distinguished SBP and DBP more clearly. The colour scheme is in keeping with EWS centile charts in use in clinical practice. We feel colour blocks are more impactful and have included an example of lines rather than colour blocks for comparison.  
 
*** Reviewer #3:  
 
Reviewer comments: 
 
*Abstract:* 
*The authors have performed a unique analysis generating gestational age-specific centiles using blood pressure data from a population-based study including women from across the world.  Their results are important as they provide information on international standards. Moreover, these results provide insight into physiological blood pressure changes in pregnancy.  Overall,  this is a clearly designed and very well-written study and the fact that it is prospective adds value to the data. The study uses strong statistical methods and analyses.*  
 
*I only do have some minor comments:* 
 
*Minor:* 
*-       Abstract:*  
*Results section: Systolic blood pressure: …. , difference (95% CI) 8.1 (7.4 to 8.8) mmHg. This sentence is unclear. At least add the word "a" as is written in the results section. However, the authors preferably state more clearly only a difference in systolic blood pressure of 8.1 mmHg (95% CI:  7.4-8.8)  between 12 weeks' gestation and 40 weeks' gestation is found.*

Thank you – for brevity we have changed the sentence to “a difference of 8·1 (95% CI 7·4 to 8·8)mmHg” as the 12 and 40 week points are already in the prior sentence. 
 
-       *Description results:*   
*I suggest to adapt the description of the results to: Median (then value) and subsequent (95% CI: values) which is easier to read and then understand the results.*

Thank you we have made this change throughout. 
 
*-       Conclusions:*   
 *The conclusion that these data provide a strong basis for constructing… (line) is not supported by these data. Hence, in the abstract the authors should use describe it as accordingly in the main paper-discussion: (Line 480-482): These gestational age-specific centiles should help build an….* 
*-*        
Removed – see above.

*Results:* 
*Line 355: minimum to maximum difference. The authors should also refer to and add the gestational age then: is it second to third trimester?*

Thank you, added. 
 
*Discussion:* 
*Interpretation:* 
*Line 408-409: These findings should aid clinicians in recognizing when a pregnant woman is becoming unwell.*   
*I disagree with this sentence as these findings as for now only help to recognize deviations from normal values.*  
*The authors should change this interpretation of their data as they have not investigated that their values aid in recognizing when a pregnant woman is becoming unwell.*  
*Furthermore, the phrase becoming unwell is too vague. They should state more specifically what they mean by this word.* 
*Additionally, they should state more clearly that it still needs to be thoroughly investigated these values are of additional value for the detection of clinically ill women.  Then only,  subsequently,  as they mention in their conclusion the results will help build an MOEWS to allow earlier recognition.*

Thank you for this suggested change in wording, which we agree is clearer. We have also added a sentence to the limitations section suggest that *“further work is needed to determine how knowledge of these centiles affects the detection of deteriorating health in pregnant women”*.  
 
*Limitations:*   
*With the aim of this study to describe blood pressure patterns in a healthy pregnancy women (with an increased risk of ) developing complications are excluded.  The authors succeeded as for example a low incidence of  gestational hypertension and  preeclampsia is reported.  They furthermore demonstrated in a sensitivity analysis that exclusion of  these women did not affect their results.* 
*However, they should mention in their limitations that the strict inclusion criteria (e.g age 18-35 years, BMI < 30) as described in their methods may contribute to less generalizability of their gestational age-specific centiles for blood pressure. Specifically, taking their future conclusion into account as their results should help clinicians determine patients with abnormal blood pressure rise and falls.*

Thank you, we have added this to the limitations. 
 
*Limitations:* 
*The authors indeed mention that lack of pre-pregnancy data may disguise first-trimester changes. They however then jump to the following conclusion (line 453-454): comparison with blood pressure in early pregnancy has greater clinical applicability.*  
*I think this statement is overrated and this conclusion cannot be drawn as there was no comparison with pre-pregnancy data as these are lacking.* 
*Hence, suggest to change this sentence to:  in routine clinical practice, blood pressure in early pregnancy contributes to clinical applicability.*

Thank you. We have changed to this wording.
