# Supplementary material for: International gestational age-specific centiles for blood pressure in pregnancy from the INTERGROWTH-21st Project in 8 countries: A longitudinal cohort study
Source: PLoS Med. 2021 Apr 27;18(4):e1003611. doi: 10.1371/journal.pmed.1003611 (PMC8112691; doi:10.1371/journal.pmed.1003611)
Supplement: Attachment — Submitted filename: Response to Editors Letter Resubmission 26.3.21.docx [file pmed.1003611.s008.docx]

Nuffield Department of Clinical Neurosciences,

University of Oxford

26th March 2021

Dear Editors,

Thank you for the opportunity to further revise our manuscript. We have included a point-by-point response, below, which we are happy for you to publish.

Yours faithfully,

Dr Lauren Green

Nuffield Department of Clinical Neurosciences, University of Oxford

Professor Peter Watkinson

Nuffield Department of Clinical Neurosciences, University of Oxford

Requests from Editors:

*The current version of the paper has a notable level of text matching with a published paper (Ohuma et al) on the INTERGROWTH project, mainly in the Methods section. We ask you to reword the relevant sections of the text to reduce this.*

Thank you, we have removed text where it is possible to reference the underlying methods articles without losing essential information for the reader. We have also reworded where possible. We would be happy to consider further suggestions form the editors.

*Please make arrangements for data deposition in a suitable publicly-accessible repository, and update the data statement in your paper.*
Done

*In the title, please move "in 8 countries" before the colon.*
Done

*Please remove "only" at line 66.*
Done

*At line 68 and any other instances, we suggest rephrasing "... dropped their blood pressure" (to, e.g., "Systolic blood pressure fell in fewer ...").*

Done *Please bullet the points in the Author summary, aiming for 3 subsections of 3 points each, comprising 1-2 short sentences.*

Done *If participants' provision of informed consent is not mentioned in the Methods section, please add this.*

Done *Please adapt the label for the STROBE checklist to "S1_STROBE_Checklist" or similar and refer to it by this label in the text.*

Done *Throughout the text, please use the style "8 diverse countries", except at the start of sentences (e.g., "Eight diverse countries ...").*

Done *Please remove the information on copyright from the end of the main text (the paper will be published under a CC-BY licence).*

Done *Please remove the information on the INTERGROWTH groups from the end of the main text (this can be transferred to a Supplementary file, if you wish).*

Thank you. We have added this to the supplementary file.
